# Physical modeling of vortical cross-step flow in the American paddlefish, *Polyodon spathula*

**DOI:** 10.1371/journal.pone.0193874

**Published:** 2018-03-21

**Authors:** Hannah Brooks, Grant E. Haines, M. Carly Lin, S. Laurie Sanderson

**Affiliations:** Department of Biology, College of William & Mary, Williamsburg, Virginia, United States of America; Ludwig-Maximilians-Universitat Munchen, GERMANY

## Abstract

Vortical cross-step filtration in suspension-feeding fish has been reported recently as a novel mechanism, distinct from other biological and industrial filtration processes. Although crossflow passing over backward-facing steps generates vortices that can suspend, concentrate, and transport particles, the morphological factors affecting this vortical flow have not been identified previously. In our 3D-printed models of the oral cavity for ram suspension-feeding fish, the angle of the backward-facing step with respect to the model’s dorsal midline affected vortex parameters significantly, including rotational, tangential, and axial speed. These vortices were comparable to those quantified downstream of the backward-facing steps that were formed by the branchial arches of preserved American paddlefish in a recirculating flow tank. Our data indicate that vortices in cross-step filtration have the characteristics of forced vortices, as the flow of water inside the oral cavity provides the external torque required to sustain forced vortices. Additionally, we quantified a new variable for ram suspension feeding termed the fluid exit ratio. This is defined as the ratio of the total open pore area for water leaving the oral cavity via spaces between branchial arches that are not blocked by gill rakers, divided by the total area for water entering through the gape during ram suspension feeding. Our experiments demonstrated that the fluid exit ratio in preserved paddlefish was a significant predictor of the flow speeds that were quantified anterior of the rostrum, at the gape, directly dorsal of the first ceratobranchial, and in the forced vortex generated by the first ceratobranchial. Physical modeling of vortical cross-step filtration offers future opportunities to explore the complex interactions between structural features of the oral cavity, vortex parameters, motile particle behavior, and particle morphology that determine the suspension, concentration, and transport of particles within the oral cavity of ram suspension-feeding fish.

## Introduction

**Fig 1:**
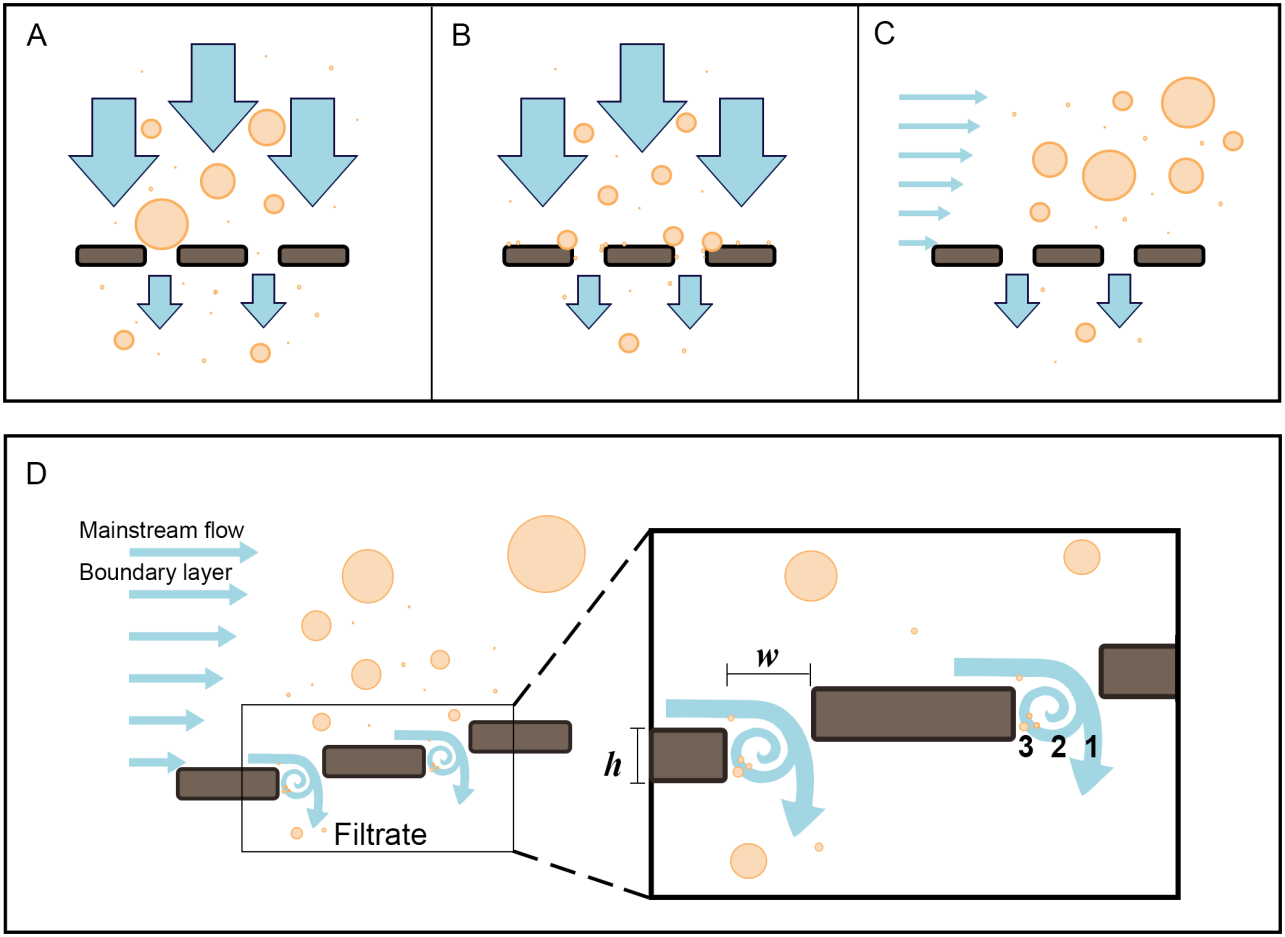
Hypothesized filtration mechanisms in suspension-feeding fishes. (A) Dead-end sieving with flow perpendicular to the filter. Small particles exit while large particles clog pores [6]. (B) Hydrosol filtration with flow perpendicular to the filter. Small particles that are retained on the sticky filter may clog pores [6]. (C) Crossflow filtration with flow parallel to the filter. Inertial lift and shear-induced diffusion cause particle migration away from the filter [7]. (D) Vortical cross-step filtration with flow approximately parallel (tangential) to the filter, which consists of backward-facing steps forming *d*-type ribs [8]. Inertial lift and shear-induced diffusion cause particle migration away from the filter. Smallest particles are concentrated and transported by vortices. Enlargement of cross-step filter (box on right), showing groove aspect ratio (*wh*^-1^, slot width divided by rib height) and zones (1, 2, 3) inside slot. Circles represent particles of different diameters, with smallest circles representing the smallest particles. Illustration by M. Carly Lin.

In the traditional view of filtration as a sieving process, particles are retained on the filter surface, ultimately resulting in clogging and a decline in performance. Suspension-feeding fishes such as carp, tilapia, and menhaden have been thought to retain food particles using branchial structures as a mechanical dead-end sieve (Fig 1A) or as a sticky hydrosol filter (Fig 1B) [1–6]. Paradoxically, however, our endoscopic studies of several pump suspension-feeding fish species and direct observations of ram suspension-feeding paddlefish have established that fouling of filtration structures by particles does not occur [7–10], and the surgical removal of filtration structures in some tilapia species does not adversely affect particle retention [11, 12]. The absence of clogging on the filtration structures in suspension-feeding fishes has ecological implications for feeding and ventilation. In addition, because clogging has severe negative impacts during industrial filtration [13, 14], studies of suspension feeding in fishes have industrial importance.

**Fig 2:**
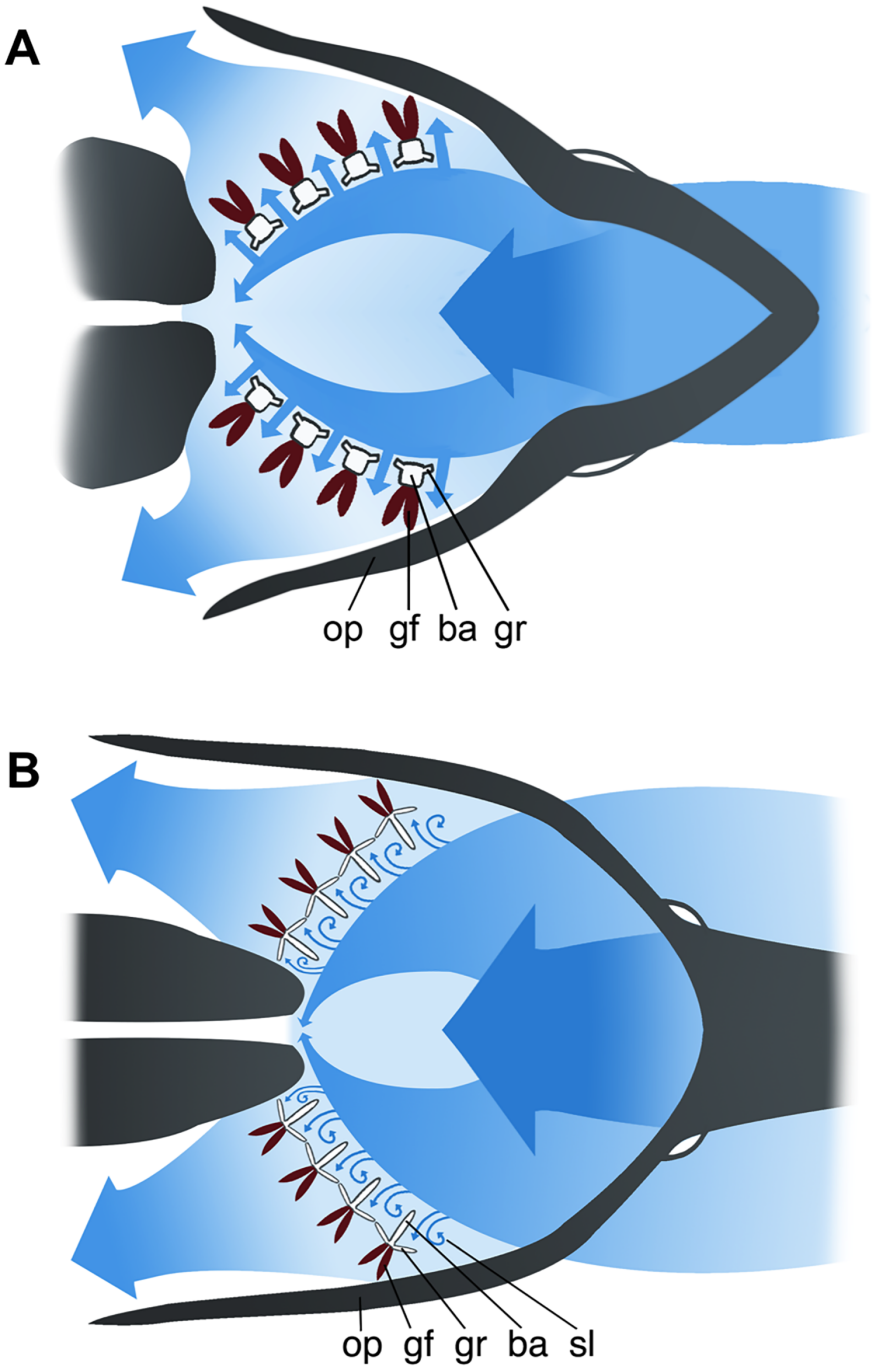
Models of fluid interaction with oral structures in suspension-feeding fishes. Coronal (frontal) sections illustrating flow patterns during (A) crossflow filtration in a species such as the blue tilapia (*Oreochromis aureus*, Cichlidae) [16], and (B) vortical cross-step filtration in the paddlefish (*Polyodon spathula*, Polyodontidae) [8]. In both (A) and (B), mainstream flow passes parallel or tangential to the filter formed by the branchial arches (ba) and the gill rakers (gr), while filtrate passes between the gill rakers and across the gill filaments (gf) for gas exchange before exiting via the opercular cavities beneath the operculum (op). In (B), the interaction of crossflow with the backward-facing steps formed by the branchial arches generates sustained vortices in the slots (sl) between the branchial arches. The first gill slot extends from the suspensorium in the oral roof and the dentary to the first branchial arch. Illustration by Virginia Greene/virginiagreeneillustration.com.

During conventional dead-end sieving, the main flow of water passes perpendicularly through the filter and particles that are larger than the mesh pore size are retained on the filter surface (Fig 1A). The discovery of crossflow filtration in fish [7, 15] (Figs 1C and 2A) shifted attention to the benefits of moving fluid parallel or tangential to a filter during suspension feeding, rather than perpendicular. Evidence for crossflow filtration has been reported in a diversity of fish species [7–10, 16–18] and balaenid whales [19, 20], and crossflow filtration has also been suggested as a likely mechanism in balaenopterid whales [19–21]. Industrial uses of crossflow filtration are extensive, including the manufacture of biotechnology products and the separation of solids from beverages and foods. In crossflow filtration, fluid dynamic processes such as inertial lift and shear-induced diffusion cause back-transport of particles away from the filter surface [7, 22]. Consequently, clogging is reduced by the transport of particles in the mainstream flow above and along the filter surface, while the filtrate passes through pores in the filter. The particles become increasingly concentrated as they are transported towards the esophagus.

While the movement of fluid parallel to the filter during industrial crossflow filtration reduces the accumulation of particles on the filter surface, clogging is still a major operating expense [13, 23]. The lack of fouling in suspension-feeding fishes indicates that hydrodynamic mechanisms in addition to crossflow filtration are involved. Vortical cross-step filtration (Figs 1D and 2B) has been reported recently as a novel crossflow filtration mechanism that can reduce clogging in suspension-feeding fishes [8]. Like crossflow filtration, cross-step filtration incorporates the hydrodynamics of parallel or tangential flow across the filter surface. However, cross-step filtration models differ from other industrial and biological filtration mechanisms by incorporating the generation of vortices as crossflow passes over unique configurations of backward-facing steps that form *d*-type ribs; *d*-type ribs are a category of roughness element in which the groove aspect ratio (width of the groove between ribs divided by rib height, Fig 1D) is less than approximately 3–4 [24, 25]. The vortices that are sustained and guided along the filter surface by this three-dimensional structure serve to clear particles continuously from the porous filter immediately downstream of each step and can transport particles within the slots between backward-facing steps [8]. In suspension-feeding fish species, the backward-facing steps that could form *d*-type ribs are (1) branchial arches, (2) gill rakers on the branchial arches, and/or (3) denticles or branchiospinules on the gill rakers. In paddlefish (*Polyodon spathula*, Polyodontidae) and basking sharks (*Cetorhinus maximus*, Cetorhinidae), thin cartilaginous branchial arches serve as *d*-type ribs, and filtrate exits through the slots between the branchial arches [8]. The extent to which structural features of the oral cavity can affect basic vortex parameters (diameter; rotational, tangential, and axial speed) during vortical cross-step filtration has not been studied. Analytical studies suggest that such parameters can affect the movement of inertial particles within the vortex [26]. In addition, these vortex parameters are indicative of velocity gradients and shear [27], which have been reported recently to have strong effects on the concentration of motile phytoplankton and zooplankton in laboratory experiments [28, 29]. Therefore, because suspension-feeding fish species consume particles including phytoplankton and zooplankton, we selected vortex parameters relating to fluid speed and direction for study.

Vortical cross-step filtration has potential ecological and economic importance beyond a reduction in filter clogging. For example, intraoral vortices could form in a diversity of suspension-feeding fish species that use crossflow filtration. Recognizing that such vortices could result in the concentration of positively buoyant particles such as oil droplets, recent experiments have demonstrated that suspension-feeding goldfish (*Carassius auratus*, Cyprinidae) can separate liquid vegetable oil from water and ingest the concentrated oil [30]. These data have toxicological implications and suggest that vortical cross-step filtration or other related crossflow filtration mechanisms might enable suspension-feeding fishes to use suspended oil droplets or a surface film of oil as a nutritional source [30].

Here, we use 3D-printed models and preserved paddlefish in a recirculating flow tank to test hypotheses on the factors affecting the operation of vortical cross-step filtration. First, we used 3D-printed models of the oral cavity in a ram suspension-feeding fish to quantify the vortex parameters (diameter; rotational, tangential, and axial speed) generated by *d*-type ribs at different angles with respect to the model midline, to test the hypothesis that branchial arch abduction and adduction can affect vortex parameters significantly. Second, we quantified vortex parameters and flow speeds in paddlefish that had been preserved in ram suspension-feeding position, to compare the flow patterns generated by the 3D models and the paddlefish specimens. Together, these experiments enabled us to test the hypothesis that the vortices in cross-step filtration have the characteristics of forced vortices, as the flow of water inside the oral cavity provides the external torque required to sustain a forced vortex. Third, we quantified a new variable for ram suspension feeding termed the fluid exit ratio. We define this variable as the ratio of the total open pore area for water to exit from the oral cavity via the spaces between branchial arches (or via the gill slits in elasmobranchs) that are not blocked by gill rakers, divided by the total area for water to enter through the gape during ram suspension feeding. We used the preserved paddlefish to test the hypothesis that the fluid exit ratio is greater than one, as this will reduce pressure drag and prevent water from being diverted in a bow wave anterior to the gape during ram suspension feeding. Tests of these hypotheses can provide insight into the evolution and energetics of ram suspension feeding, a distinctive feeding mode used by many species of economic and ecological importance (e.g., herring, anchovies, mackerel) as well as the largest extant and extinct fish species (e.g., basking sharks, whale sharks, manta rays, extinct pachycormids [1, 31, 32]).

## Methods

### 3D-printed model

#### 3D model design

The cross-step physical model design of Sanderson et al. [8] was used. Models were developed in SketchUp Pro 2014 (Trimble Navigation) and 3D-printed in nylon plastic (fine polyamide PA 2200, Shapeways). The branchial arches were modeled as solid *d*-type ribs separating open slots that simulated the internal gill slits between arches. The most anterior rib corresponded to the lower jaw and the upper jaw, including the anterior portion of the oral roof. Ribs 2 through 5 of the models corresponded to branchial arches I–IV. The solid end of the conical models represented the fifth branchial arch and the esophagus.

Because fish can abduct and adduct their branchial arches, we tested the hypothesis that vortex parameters differ significantly depending on the angle of the *d*-type rib with respect to the midline of the model roof. This angle of the backward-facing steps that formed *d*-type ribs was varied to be 55°, 90°, or 110°. The conical dimensions for the three designs were identical, including the open gape area for water to enter. We refer to the internal spaces between branchial arches as gill “slots” to emphasize that they are spaces between the branchial arches (separating the oral cavity from each of the two opercular cavities), rather than the single opercular slit leading from each opercular cavity to the exterior of the fish. The unobstructed area of the gill slots formed by the medial margins of the branchial arches, through which water exited from the models, differed among designs by < 1.3%. To simulate the gill rakers, nylon mesh (140 μm pore size, open pore area percentage of 55%, Component Supply Co.) covered the lateral margins of the ribs on the models. The total open pore area of the slots was 160% of the models’ gape.

The gill slots of the models were cut out of the wall of the cone, with the branchial arch height corresponding to the wall thickness. Thus, the arches did not project into the oral cavity. Rather, the arches formed portions of the model roof and floor, as in the oral cavity of fish. When the lower jaw abducts during ram suspension feeding, the oral cavity forms an asymmetrical cone. To incorporate this asymmetry into the models, the dorsal half of the cone in each model was scaled by a factor of 0.6 and the ventral half was scaled by a factor of 1.1 in SketchUp Pro 2014. At the roof of each model, the height of the arches was 3.7 mm. This height increased gradually towards the floor of the model, where the height of the arches was 6.7 mm. The height of the arches (*h*) was comparable to the anterior to posterior width of the slots (*w*) between arches (6.0 mm), with an aspect ratio (*wh*^*-1*^, Fig 1D) ranging from 0.9 to 1.6 [8].

#### 3D model vortex experiments

Each model was positioned in the center of a recirculating flow tank (18 x 18 x 90 cm working area, 100 l total volume) parallel to the oncoming flow so that water entered through the open gape of the model and exited through the mesh covering the slots between the simulated branchial arches. The models were suspended using a sting attached to the closed posterior end of the model. A transparent plastic skirt was attached externally along the anterior region of the model to simulate the paddlefish operculum. Rhodamine dye visualization was used instead of digital particle image velocimetry (DPIV) because the vortices traveled along their axes within the slots between the deep ribs of the model, medial to the mesh that simulated the gill rakers. The deep ribs and the mesh block laser illumination, precluding the use of DPIV in these models. A polyethylene cannula (1.14 mm I.D., 1.57 mm O.D., Intramedic PE-160) was inserted into a 1.59 mm hole drilled through the solid midline at the top of each model. The tip of the cannula was positioned to be close to the model wall at the dorsal end of the second slot, corresponding to the slot between the first and second branchial arches in the preserved paddlefish. The cannula was connected to a syringe outside the flow tank, and rhodamine water-tracing dye (Cole Parmer) was released gradually into the second slot between the ribs of the model to visualize and record the vortices (120 frames s^-1^, Sony RX10M2 and Apple iPhone 5S). Each of the models had a millimeter scale on an acetate sheet glued to be flush with the downstream exterior of the rib that was immediately anterior to the slot in which the vortices were recorded. The speed of the water recirculating in the flow tank was maintained at 18.4 ± 0.4 (mean ± SD) cm s^-1^, using a motor and adjustable speed control (Bodine Electric Co. models 24A4BEPM and 815, respectively) attached to an aluminum impeller. The flow speed was measured using a Geopacks MFP51 flowmeter impeller positioned in the center of the flow tank, when the model was absent from the flow tank.

The recordings of the vortices were analyzed using iMovie ‘11 (version 9.0.4). Five vortices were analyzed frame-by-frame for each model angle. Each clip was paused when the vortex had completed the maximum number of full 360° revolutions (*R*) observable in the field of view. The number of frames needed for the vortex to complete these full revolutions was converted to the time elapsed. Images of the vortex and the millimeter ruler along the rib were analyzed in ImageJ 1.49 (National Institutes of Health) to quantify the vortex diameter and the distance traveled by the vortex along the vortex axis within the slot between ribs. For each of the vortices, these values were used to calculate the angular velocity, rotational speed, tangential speed, and speed of the vortex along its axis within the slot between ribs.

The rotational speed of the vortex (*S*) was expressed in revolutions min^-1^ by dividing the number of revolutions that the vortex completed (*R*) by the time taken to complete those rotations (*t*, s), and multiplying by 60 s min^-1^.

$$S=(\frac{R}{t})(\frac{60\;s}{min})$$

Angular velocity of the vortex (Ω, s^-1^) was calculated by converting the number of 360° revolutions (*R*) to radians and dividing by time required (*t*, s) to complete that number of revolutions.

$$\Omega =\frac{R(2\pi )}{t}$$

The linear flow speed along the circular path of the vortex (tangential speed, *V*, cm s^-1^) was calculated by multiplying the angular velocity (Ω, s^-1^) by the radius of the vortex (*r*, cm).

V=Ωr

The speed at which the vortex advanced along its axis as the vortex traveled helically within the slot between ribs (*A*, cm s^-1^) was calculated by dividing the distance traveled by the vortex along the slot during *R* revolutions (*d*, cm) by the time it took to travel that distance (*t*, s).

$$A=\frac{d}{t}$$

#### 3D model particle concentration patterns

Using the flow tank as described above, a separate set of experiments was conducted to visualize effects of the vortices on patterns of particle concentration in the models. For this purpose, 0.60 g of brine shrimp cysts was hydrated (*Artemia*, 210–300 μm diameter, density 1.09 g cm^-3^, 10 ppm volume concentration) and added to the flow tank. Photographs were taken after each model had retained particles for 3.0 min.

### Preserved paddlefish

#### Paddlefish specimens

Juvenile paddlefish (32.5–45.5 cm total length, TL; n = 3 fish) were obtained on ice within 24 h of death from an aquaculture facility (William and Mary Institutional Animal Care and Use Committee approval 07/30/14; Virginia Department of Game and Inland Fisheries approval 07/24/14). Specimens were designated as A (eye-fork length, EFL = 18.0 cm), B (EFL = 19.0 cm), and C (EFL = 29.0 cm). Paddlefish were preserved in 10% buffered formalin with gauze sponges placed between the branchial arches to fix the oral cavity in ram suspension-feeding position [8]. Preserved specimens were stored in 75% ethanol.

#### Paddlefish vortex experiments

Each paddlefish preserved in suspension-feeding position was mounted in the center of the flow tank using an overhead clamp that was flush with the dorsal body surface downstream from the opercular cavities. To account for the maximum yaw angle of 6.1° ± 2.1° (mean ± SD) quantified recently in live paddlefish [33], the preserved paddlefish were angled slightly to the right side of the flow tank, so that the midline of the anterior oral cavity in each specimen (n = 3 fish) was angled at 4.5° ± 1.8° with respect to the direction of oncoming flow. The speed of the water recirculating in the flow tank was maintained at 18.9 ± 0.2 cm s^-1^ for all paddlefish experiments. This speed is at the low end of the range reported for swimming speeds in live paddlefish of approximately the same body length [34].

During feeding in paddlefish, the gill rakers abduct to cover the floor of the slots between branchial arches. During ram ventilation and after death, the gill rakers adduct passively to lie vertically against the branchial arches. In the preserved paddlefish, the gill rakers in the slot between the first and second branchial arches on the left side of each paddlefish were simulated using a stainless steel mesh (104 μm pore size, open pore area percentage of 35%, Ted Pella Inc.). To prepare the mesh, tracings of the slots were used as a template to cut the mesh to the correct sizes and shapes. The mesh then fit tightly along the lateral margins of the slots, where the gill rakers of paddlefish are attached to the branchial arches.

To visualize the vortices, an infusion needle (25G x 3/4”, 30 cm tubing, Terumo Medical Corporation) was inserted through the wall of the first branchial arch on the left side of each paddlefish. The needle was inserted from a downstream location that was ventrolateral and external to the fish oral and opercular cavities. The needle tip was flush with the posterior wall of the first ceratobranchial at approximately the junction between the first ceratobranchial and the first hypobranchial, and was pointed into the slot between the first and second branchial arches. This minimized the needle’s effect on the flow patterns between the arches, as the needle did not project between the first and second arches where the vortices were recorded. A syringe with rhodamine was attached to the infusion needle tubing external to the fish, downstream and ventrolateral from the oral cavity. A thin stream of dye was released gradually from the tip of the infusion needle into the slot between arches while water was flowing through the paddlefish oral cavity, allowing visualization and recording of the flow patterns between the arches (120 frames s^-1^). While continuing to record from the same position, a millimeter ruler was then placed along the arch of the paddlefish in the location of the flow patterns to provide a scale for analysis.

Five vortices in each paddlefish specimen were analyzed using the same procedure as that applied to the 3D models. Angular velocity, rotational speed, tangential speed, and speed of the vortex along its axis as the vortex traveled within the slot between branchial arches were calculated using the same equations as listed above for the vortices in the 3D models.

#### Paddlefish flow speed experiments

To measure flow speed anterior of and inside the oral cavity of the preserved paddlefish, a probe with a glass bead thermistor (1.09 mm diameter, 112-101BAJ-01, Fenwal Electronics) was connected to a circuit modified from LaBarbera and Vogel [16, 35]. The output of the circuit was sampled at 200 Hz by a Sonometrics TRX-4 A/D convertor and displayed in real time. The probe was threaded through a polyethylene cannula (1.57 mm I.D., 2.08 mm O.D., Intramedic PE-205) until the glass bead protruded slightly from the tip of the cannula and a flow speed maximum was recorded. Digitized recordings (125 frames s^-1^, Intensified Imager VSG, Kodak) of rhodamine dye that was released 3 cm anterior of the tip of the paddlefish rostrum were used to confirm the corresponding values from the A/D convertor.

The cannula with the thermistor flow probe was positioned to record flow speed (a) 3 cm anterior of the rostrum tip, (b) immediately anterior to the gape at a height directly dorsal to the first ceratobranchial, and (c) inside the oral cavity directly dorsal to the first ceratobranchial, above the insertion site of the infusion needle. These flow speed measurements were obtained at the conclusion of each paddlefish vortex experiment, prior to moving the paddlefish or the infusion needle. The speed of the water recirculating in the flow tank was maintained at 18.9 ± 0.2 cm s^-1^ for all paddlefish experiments.

#### Ratio of slot area between paddlefish branchial arches to oral gape area

**Fig 3:**
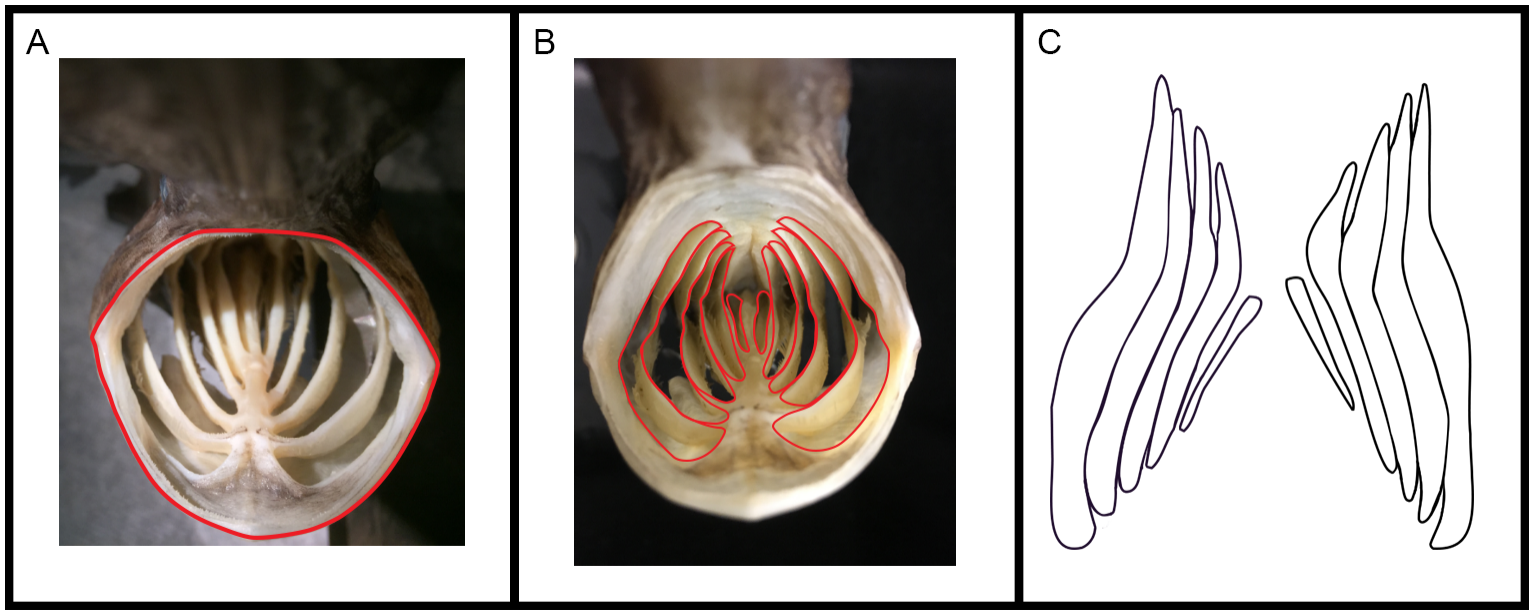
Methods to quantify gape area and gill slot area in preserved paddlefish. Paddlefish that had been preserved in suspension-feeding position were held as if mounted in the flow tank (A) and ImageJ 1.49 was used to quantify the area inside the perimeter of the oral gape (outlined in red). The medial edges of structures along the margins of the gill slots (schematic shown in B) were then traced by hand onto a clear vinyl sheet that had been inserted inside the oral cavity to be flush with these structures. This method reproduced the outlines of the slots as a two-dimensional image (C), and ImageJ was then used to quantify the area of the gill slots. Illustration by M. Carly Lin.

To quantify the oral gape area, each paddlefish (n = 3 fish) was positioned as if mounted in the flow tank and was photographed with a millimeter ruler in anterior view, ensuring that the rostrum did not obscure any portion of the oral gape (Fig 3A). ImageJ 1.49 was used to quantify the two-dimensional area inside the perimeter of the gape (Fig 3A). To quantify the slot area for each paddlefish preserved in ram suspension-feeding position, the medial edges of the ceratohyal, branchial arches, basibranchials, and the cartilaginous element posterior of the fifth ceratobranchials (“uga” of Grande and Bemis [36]) were traced by hand onto a clear vinyl sheet (20 gauge) that had been inserted inside the oral cavity and was flush with the medial edges of these structures. By tracing these structures onto the vinyl sheet that had been placed inside the oral cavity, the outlines of the gill slots (Fig 3B) were reproduced as a two-dimensional image (Fig 3C). To compare the oral gape area with the slot area between branchial arches, the area of each slot between consecutive branchial arches through which water exits from the oral cavity (Fig 3C) was quantified from the tracings in ImageJ 1.49. Because the gill rakers are adducted against the walls of the branchial arches in dead paddlefish, the area blocked by gill rakers during suspension feeding was not excluded from this quantification of slot area.

#### Paddlefish branchial arch angles

To quantify the angles at which branchial arches I–IV were arranged along the midline of the oral cavity roof and floor in the paddlefish preserved in suspension-feeding position, an acetate sheet was cut to fit tightly into the dorsal or ventral half of the paddlefish oral cavity. Tracings were made along the medial edge of each epibranchial and infrapharyngobranchial in the oral cavity roof, as well as the suspensorium forming the anterior wall of the first gill slot in the oral roof. In addition, each ceratobranchial, hypobranchial, and basibranchial in the oral cavity floor was traced, as well as the lower jaw. The suspensorium in the oral roof and the dentary were included in the measurements of branchial arch angles because these structures form the anterior margin of the first gill slot, while the first epibranchial and the first ceratobranchial form the posterior margin of the first gill slot.

The tissue covering the infrapharyngobranchials in the oral roof and the basibranchials in the oral floor formed a symmetrical wedge centered along the midline of the roof or floor, with the point of the wedge facing the posterior oral cavity. Therefore, a line was drawn along the straightest region of each epibranchial and each ceratobranchial, and each line was extended to meet the wedge formed by the infrapharyngobranchials or the basibranchials. The angle at which each extended epibranchial or ceratobranchial intersected this wedge was quantified using ImageJ 1.49. For each branchial arch in each paddlefish, the angles from the right and left sides of the oral cavity were averaged together.

### Statistical analysis

RStudio (Mac v 1.0.136) with R (v 3.3.3) was used for linear regression to model flow speed as a function of fluid exit ratio. JMP 12 Mac (SAS Institute Inc.) was used for all other statistical tests. Using the sequential Bonferroni method of Holm [37, 38], the probability of a Type I error was less than an α value of 0.05 for the family consisting of all ANOVAs performed in the study. Levene’s tests for homogeneity of variance were performed. To test for differences in vortex revolutions per minute, tangential speed, diameter, and speed along the vortex axis among the 3D models with ribs at different angles (55°, 90°, or 110°), one-way ANOVAs were used only when the variances were homogeneous. These statistical tests were also used to compare the vortices among the preserved paddlefish specimens. Angular velocity was not tested statistically, because both angular velocity and rotational speed rely on the same measurements. When the sequential Bonferroni method indicated significant differences between groups in the one-way ANOVAs, Tukey’s honest significant difference (HSD) *post hoc* test was performed.

## Results

### 3D model vortex experiments

**Fig 4:**
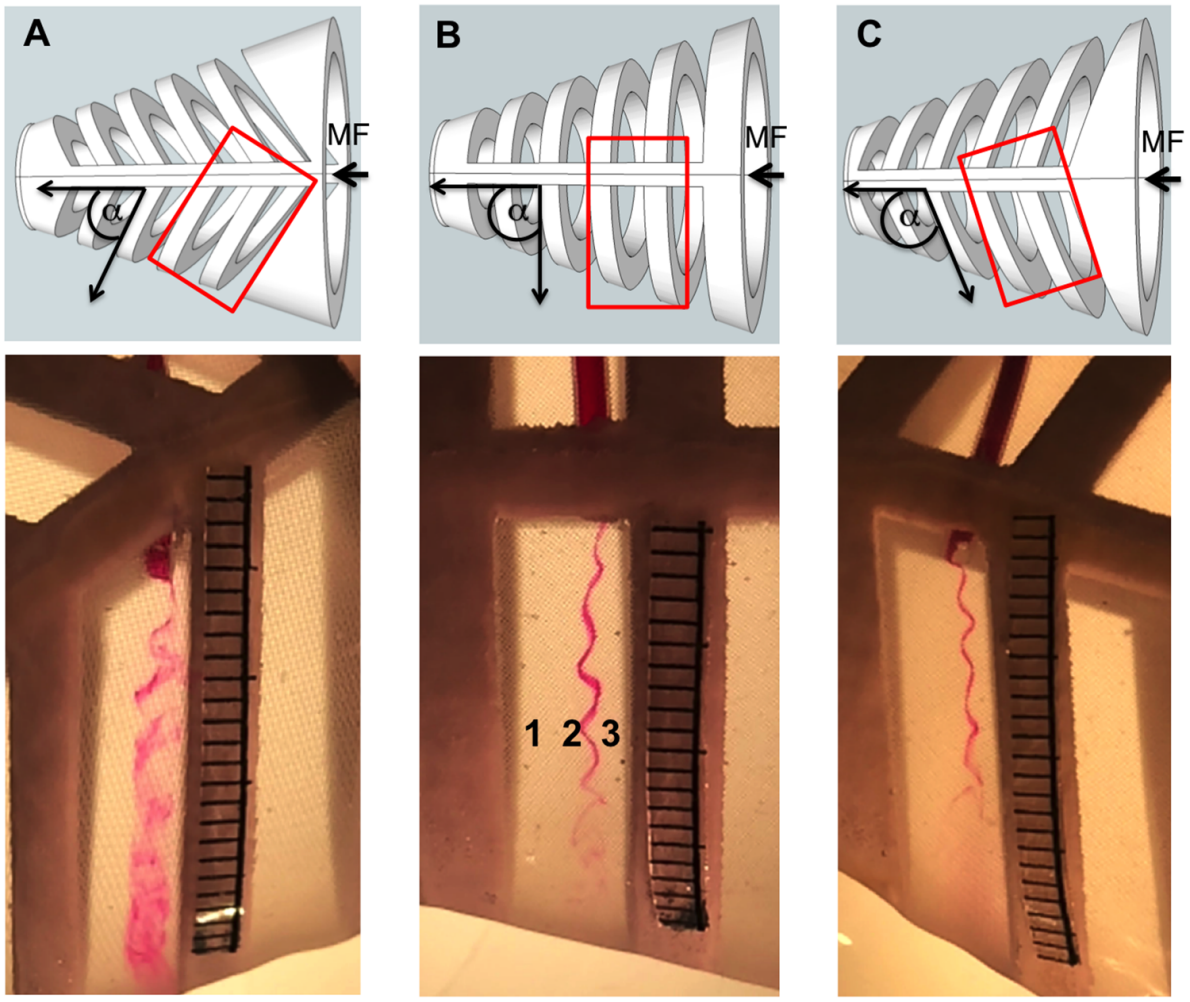
Sustained vortices were generated in 3D models. Dorsal views of the computer-aided design (CAD) images (top) and 3D-printed models (bottom) in the recirculating flow tank, with mainstream flow (MF) entering the open gape at the right of each image. Red rectangles on CAD images denote locations on 3D models where vortices were recorded for analysis. Zones (1, 2, 3) are labeled inside the slot as in Fig 1D. The sustained vortex that formed in zone 2 was visualized by introducing dye into the slot between the second and third ribs via a cannula inserted through the dorsal midline of each model. The mesh that covered the lateral margins of the slots in all models is most visible in (A). (A) α = 55°, (B) α = 90°, (C) α = 110°. Scale in mm.

**Fig 5:**
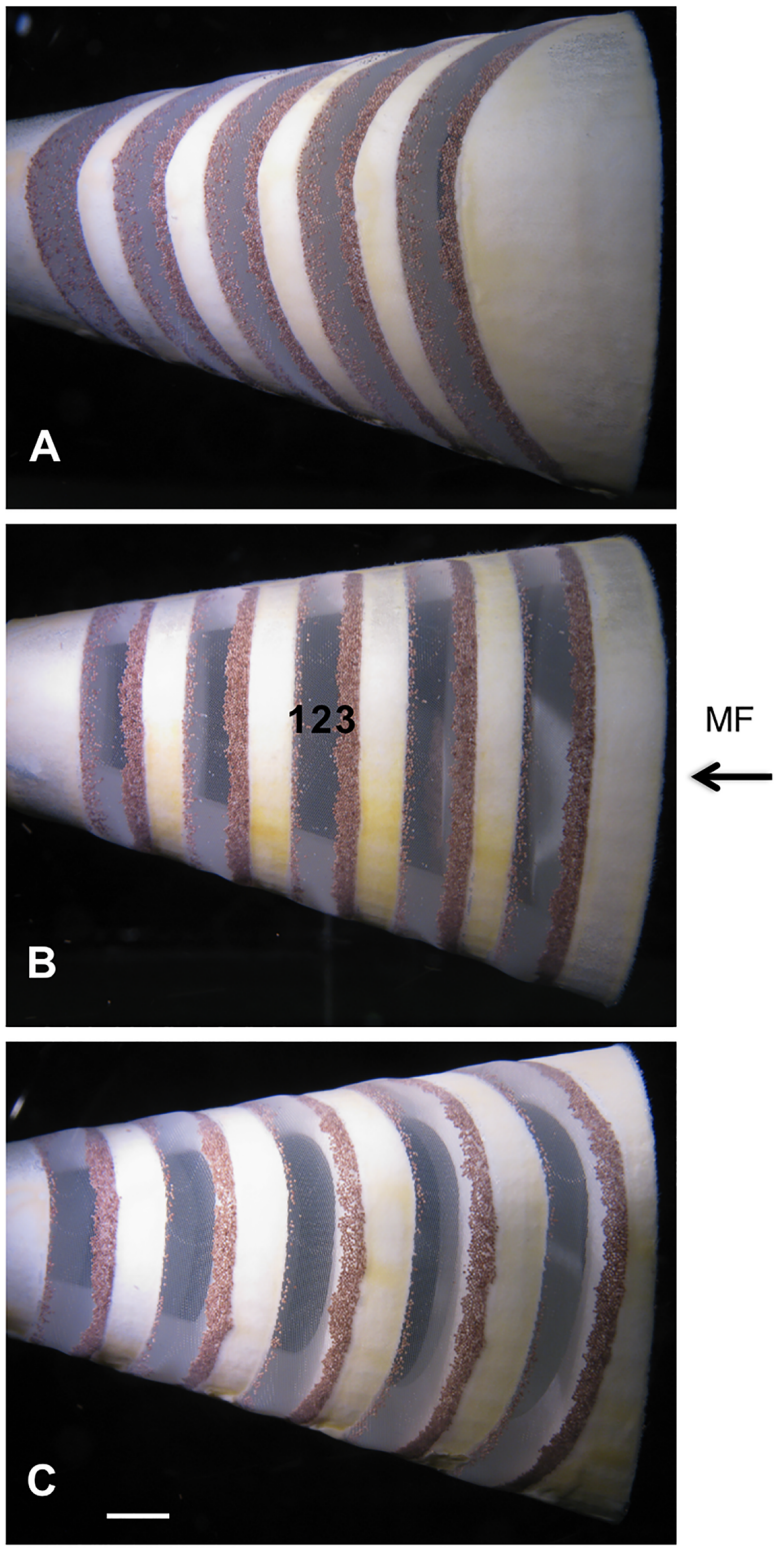
Vortices caused particle concentration along slot margins in 3D models. Lateral views of 3D models in the recirculating flow tank, with mainstream flow (MF) entering the open gape at the right of each image. Particles (*Artemia* cysts, 210–300 μm diameter) were concentrated in zones 1 and 3 of all models, while vortices scoured the particles from the mesh in zone 2. (A) 55° model, (B) 90° model, (C) 110° model. Scale bar 0.5 cm. Photos by Pablo Yañez.

In the three models that had different rib angles (55°, 90°, or 110° with respect to the midline of the model roof), vortices were generated continuously as fluid passed over the backward-facing steps that formed the *d*-type ribs. Due to the sudden expansion of volume in the slot immediately downstream from each rib, a recirculation of fluid occurred in zone 2 of each slot (Figs 4 and 1D). In addition, fluid passing directly over the medial surface of the rib then separated from the medial downstream edge of the rib as a shear layer that entered the slot in zone 2 [8]. In each slot, this separated shear layer wrapped around the recirculation region in zone 2, scouring particles from zone 2 and transporting the particles to zone 3. This effectively prevented clogging of the mesh in zone 2 (Figs 5 and 1D). Farther downstream in each slot, the fluid that passed medial to the separated shear layer then entered the slot and exited from the slot through the mesh in zone 1, where some particles were deposited (Figs 5 and 1D).

The vortical flow created by the recirculation region and the separated shear layer in zone 2 traveled helically along the vortex axis within the slot. The dorsoventrally asymmetrical shape of the conical model, combined with the asymmetrical plastic skirt that was attached externally along the anterior region of the model to simulate the paddlefish operculum, caused more water to exit from the slots near the ventral midline of the model rather than the dorsal midline of the model. This greater exit of water from the ventral areas of the model established the dorsal to ventral movement of both the recirculation region and the separated shear layer as a helical flow within each slot.

Diameters of the vortices varied significantly among the three models (Table 1, one-way ANOVA, *p* < 0.0001, n = 5 vortices). The 90° model and the 110° model had vortices with a mean diameter of 0.09 cm, while the vortices in the 55° model differed significantly from both of them (*p* < 0.0001, Tukey HSD *post hoc* tests) with a mean diameter of 0.21 ± 0.02 cm. The greater mean diameter that was measured for the vortices in the 55° model resulted from the mixing of dye near the core of the recirculation region with fluid from the outer margins of the vortex, indicating unsteady rotational flow (Fig 4A). Despite this disorganized flow in the 55° model, the separated shear layer continued to wrap around the recirculation region in zone 2 to generate a cohesive rotational flow that traveled helically along the vortex axis within each slot. As in the 90° model and the 110° model, this vortical flow in the 55° model was effective in scouring the mesh in zone 2 and preventing clogging (Fig 5).

**Table 1:** Vortex parameters quantified for 3D cross-step models (mean ± SD, n = 5 vortices).

Angle of Model	Diameter (cm)	Rotational Speed (revolutions min^-1^)	Tangential Speed (cm s^-1^)	Speed along axis in slot (cm s^-1^)
**55°**	0.21 ± 0.02	535.8 ± 51.7	5.8 ± 0.7	3.3 ± 0.1
**90°**	0.09 ± 0.02	607.5 ± 45.3	3.0 ± 0.8	3.0 ± 0.4
**110°**	0.09 ± 0.00	943.4 ± 149.0	4.5 ± 0.7	3.8 ± 0.2

The rotational speeds of the vortices in the three models also varied significantly (*p* < 0.0001, one-way ANOVA, n = 5). The vortices in the 110° model had the fastest mean rotational speed compared to the 55° model and 90° model (Table 1, *p* < 0.0001 and *p* = 0.0008 respectively, Tukey HSD *post hoc* test). Tangential speeds of the vortices varied significantly among the three model angles (one-way ANOVA, *p* < 0.0002, n = 5). The vortices in the 90° model had a significantly slower mean tangential speed compared to the 55° and 110° models (*p* = 0.0002 and *p* = 0.02 respectively, Tukey HSD *post hoc* tests), while the vortices in the 110° model had a significantly slower mean tangential speed than the 55° model (*p* = 0.04, Tukey HSD *post hoc* test). The speed at which the vortex traveled along its axis within the slot varied significantly between the models (one-way ANOVA, *p* = 0.0009, n = 5). The 110° model vortices traveled at a greater speed within the slot compared to the vortices in the 55° and 90° models (*p* = 0.001 and 0.0008, respectively, Tukey HSD *post hoc* test).

### Paddlefish vortex experiments

**Fig 6:**
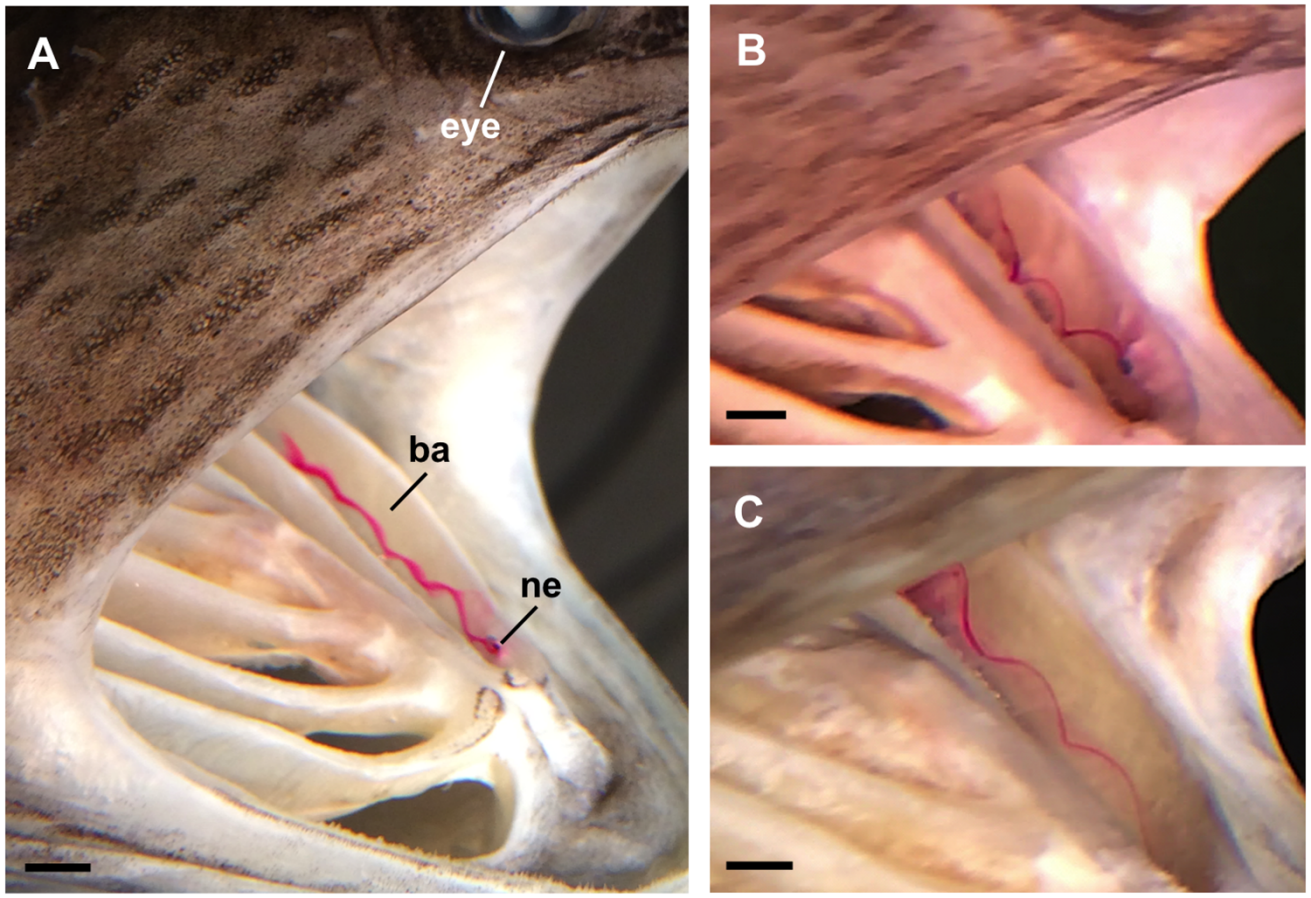
Sustained vortices were generated inside the oral cavity of paddlefish that had been preserved in ram suspension-feeding position. Lateral views of three paddlefish specimens (A, B, C) in the recirculating flow tank, with mainstream flow entering the open gape at the right of each image. Vortices were visualized by introducing dye into the slot between the first and second ceratobranchials via the tip of an infusion needle (ne) that was flush with the first branchial arch (ba). Scale bars 0.5 cm.

In the preserved paddlefish, the rhodamine dye from the infusion needle allowed visualization of the sustained vortices that were generated as flow entered the oral cavity and passed over the backward-facing step formed by the first ceratobranchial (Fig 6). The vortices traveled along the slot between ceratobranchial I and ceratobranchial II, moving continuously in a helical path towards the ceratobranchial-epibranchial junction, where vortex breakdown occurred and the dye dispersed into the opercular cavity.

The vortex diameters and the tangential speeds were not significantly different among the preserved paddlefish (Table 2, one-way ANOVA, *p* = 0.06, n = 5 vortices). However, the rotational speeds of the vortices were significantly different (one-way ANOVA, *p* < 0.0001, n = 5), with the vortices in Paddlefish A having the highest mean rotational speed compared to the other paddlefish (*p* < 0.0001, Tukey HSD *post hoc* tests). In addition, the vortices in the three paddlefish traveled at significantly different speeds along the vortex axis in the slot between the first and second ceratobranchials (one-way ANOVA, *p* = 0.0005, n = 5).

**Table 2:** Vortex parameters quantified for paddlefish preserved in suspension-feeding position (mean ± SD, n = 5 vortices).

Paddlefish Specimen	Diameter (cm)	Rotational Speed (revolutions min^-1^)	Tangential Speed (cm s^-1^)	Speed along axis in slot (cm s^-1^)
**A**	0.19 ± 0.04	641.2 ± 42.1	6.4 ± 1.4	5.1 ± 0.4
**B**	0.26 ± 0.06	398.7 ± 39.5	5.5 ± 1.5	4.1 ± 0.8
**C**	0.23 ± 0.02	359.7 ± 26.2	4.3 ± 0.7	6.3 ± 0.5
**Mean ± SD**	0.23 ± 0.04	466.5 ± 152.5	5.4 ± 1.1	5.2 ± 1.1

### Paddlefish flow speed experiments

The flow speed decreased as the flow probe was moved from (a) 3 cm anterior of the rostrum tip, to (b) immediately anterior of the gape at a height directly dorsal of the first ceratobranchial, to (c) inside the oral cavity directly dorsal of the first ceratobranchial (Table 3). The flow speed recorded directly dorsal of the first ceratobranchial inside the oral cavity was approximately 60–85% of the speed that was recorded anterior of the rostrum tip in the recirculating flow tank. For comparison, the tangential speed of the vortices recorded in the slot between the first and second ceratobranchials of the preserved paddlefish (Table 2) was approximately 55% of the flow speed recorded directly dorsal of the first ceratobranchial, above the insertion site of the infusion needle in the wall of the first ceratobranchial (Table 3). The Reynolds number (Re) for flow entering the gape of the preserved paddlefish, calculated using the hydraulic diameter of the gape (3.9 ± 0.7 cm, n = 3 fish), was 4200 ± 500. The Re for flow over the first branchial arch, calculated using the height of the first ceratobranchial in the region where the infusion needle had been inserted (4.4 ± 0.6 mm, n = 3 fish), was 420 ± 30.

**Table 3:** Flow speeds in the oral cavities of paddlefish preserved in suspension-feeding position (mean ± SD, n = 3 recordings per paddlefish per location).

Paddlefish Specimen	Flow Probe Location		
	3 cm anterior of rostrum (cm s^-1^)	Immediately anterior of gape at height of first ceratobranchial (cm s^-1^)	Directly dorsal of first ceratobranchial (cm s^-1^)
**A**	13.2 ± 0.4	11.8 ± 0.2	11.1 ± 0.3
**B**	12.4 ± 0.2	10.5 ± 0.3	10.2 ± 0.1
**C**	12.4 ± 0.3	9.7 ± 0.3	7.9 ± 0.2
**Mean ± SD**	12.7 ± 0.3	10.7 ± 0.6	9.7 ± 0.1

### Ratio of slot area between paddlefish branchial arches to oral gape area

The internal gill slit between the first branchial arch, the lower jaw and suspensorium along the oral floor, and the suspensorium along the oral roof, was labeled as slot 1. The slot areas between branchial arches decreased steadily from anterior to posterior (Table 4). As the fifth branchial arch lacks epibranchials, the most posterior slot was restricted to the gap between the ceratobranchials of the fourth and fifth branchial arches. Therefore, the area of slot 5 was substantially smaller than the areas of the other slots. For the three paddlefish specimens preserved in suspension-feeding position, the sum of the slot areas between arches ranged from approximately 150–190% of the oral gape area (Table 4). However, this quantification of slot area does not exclude the area blocked by gill rakers that are abducted during suspension feeding, and is therefore larger than the total open pore area between branchial arches in live suspension-feeding paddlefish.

**Table 4:** Paddlefish gill slot area and oral gape area (cm^2^).

Location	Paddlefish Specimen		
	A	B	C
**Gill Slot 1**	6.48	5.29	11.63
**Gill Slot 2**	4.30	5.51	8.59
**Gill Slot 3**	3.42	3.82	5.90
**Gill Slot 4**	2.47	2.27	5.24
**Gill Slot 5**	0.52	0.78	1.39
**Sum Gill Slots 1–5**	17.19	17.67	32.75
**Gape**	11.45	10.48	17.54
**Ratio of gill slot area to gape area**	1.50	1.69	1.87

**Fig 7:**
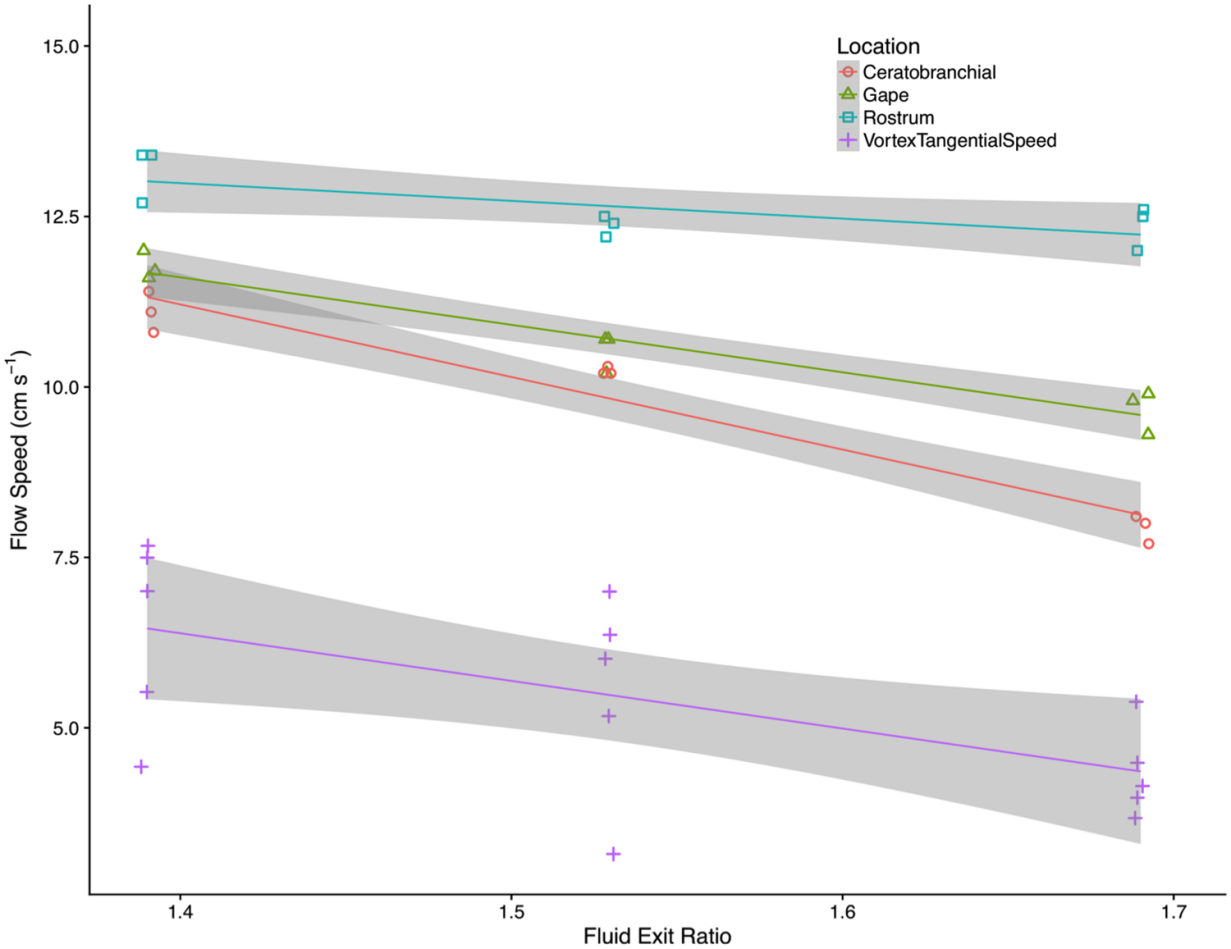
Fluid exit ratio was a significant predictor of flow speed in preserved paddlefish. Linear regressions showed significant relationships between the fluid exit ratios for the three preserved paddlefish that were used in the flow tank experiments and the flow speeds anterior of the rostrum (R^2^ = 0.49, adj. R^2^ = 0.42, *p* = 0.035, n = 3 recordings per fish), at the gape (R^2^ = 0.92, adj. R^2^ = 0.91, *p* < 0.0001, n = 3 recordings per fish), and directly dorsal of the first ceratobranchial (R^2^ = 0.94, adj. R^2^ = 0.93, *p* < 0.0001, n = 3 recordings per fish), as well as the tangential speed of the forced vortex generated by the first ceratobranchial (R^2^ = 0.37, adj. R^2^ = 0.33, *p* = 0.02, n = 5 vortices per fish). Shaded regions represent 95% confidence intervals of each regression line. Horizontal jitter was applied for better visibility of the points, and does not represent variation in fluid exit ratio within fish.

The ratio of gill slot area to gape area in the preserved paddlefish (Table 4) was then adjusted to exclude the slot area blocked by the mesh that was used to simulate the gill rakers in the flow tank experiments. The resulting fluid exit ratios for the preserved paddlefish in the flow tank experiments were a significant predictor (Fig 7) of the flow speeds that were recorded using a thermistor flow probe placed anterior of the rostrum (Table 3), at the gape (Table 3), and directly dorsal of the first ceratobranchial (Table 3). The fluid exit ratios for the preserved paddlefish were also a significant predictor (Fig 7) of the tangential speed that was calculated in the forced vortex generated by the first ceratobranchial (Table 2).

### Paddlefish branchial arch angles

For the paddlefish that had been preserved in suspension-feeding position, the mean angle between the straightest section of the anterior wall of the first gill slot on the oral cavity roof and the wedge of tissue formed by the infrapharyngobranchials along the roof midline was 46.4° ± 5.4° (n = 3 fish, Table 5). The corresponding mean angle between the straightest section of the dentary and the wedge of tissue formed by the basibranchials along the midline of the oral floor was 41.9° ± 4.0°. The mean angles between the extended epibranchials I–IV and the infrapharyngobranchials on the oral roof ranged from 41.4°–18.3°, while the mean angles between the extended ceratobranchials I–IV and the basibranchials on the oral floor ranged from 26.1°–15.0°. The angles at which branchial arches I–IV were arranged along the midline of the oral cavity roof and floor were consistently larger at the anterior of the paddlefish oral cavity and decreased with each successive arch. However, from arches I to IV, the epibranchial angles decreased more rapidly than the ceratobranchial angles (Table 5).

**Table 5:** Paddlefish branchial arch angles (mean ± SD, n = 3 fish).

Location	Angle (degrees)
**Roof**	46.4 ± 5.4
**Epibranchial 1**	41.4 ± 4.5
**Epibranchial 2**	35.4 ± 5.0
**Epibranchial 3**	33.9 ± 4.6
**Epibranchial 4**	18.3 ± 7.1
**Dentary**	41.9 ± 4.0
**Ceratobranchial 1**	26.1 ± 2.6
**Ceratobranchial 2**	20.7 ± 3.3
**Ceratobranchial 3**	18.8 ± 5.0
**Ceratobranchial 4**	15.0 ± 5.0

## Discussion

Vortical cross-step filtration is distinct from other biological and industrial filtration mechanisms because this mechanism uses backward-facing steps that form *d*-type ribs on the porous surface of a cone [8]. The *d*-type ribs reduce clogging by manipulating the shear layer and generating organized patterns of vortices that can suspend, concentrate, and transport particles along the filter surface. These vortices were identified recently as hydrodynamic features that are generated when crossflow interacts with three-dimensional branchial structures in suspension-feeding fish. Sanderson et al. [8] reported that the vortices in the second gill slot of paddlefish that had been preserved in suspension-feeding position resulted in the concentration of brine shrimp cysts in zone 3 of the slot, directly downstream from the first ceratobranchial. While previous research noted a number of possible roles for vortices during fish suspension feeding [7, 9, 16, 39, 40], the existence and function of vortical cross-step filtration had not been identified previously.

Industrial applications of crossflow filtration typically use high transmembrane pressures (30–200 kPa) to force fluid through filters that have pore sizes < 1 μm [41]. Consequently, the Reynolds number (Re) for filtrate flow through the pores of industrial crossflow filters is typically << 1. Under these conditions, the three-dimensional features of the filter pores do not generate vortices inside the pores. In contrast to many industrial filtration systems, the available data indicate that suspension-feeding fishes use larger filter pore sizes, resulting in Re values of approximately 10–500 for flow through the filter pores [7, 17, 18, 42, 43]. In ram suspension-feeding fishes, the minimal pressure drops that have been estimated across the filter (approximately 10–150 Pa from the interior of the oral cavity to the opercular cavities [8, 17, 44]), combined with larger pore sizes that result in higher Re at the level of the pore, have the potential to result in vortex formation at the level of the filter pores along (1) the branchial arches, (2) the gill rakers on the branchial arches, and/or (3) the denticles or branchiospinules on the gill rakers.

Some industrial crossflow systems incorporate three-dimensionality into either the design of inserts or the surface of the filter membrane (e.g., corrugations, baffles, constrictions, spirals [45–48]). The objective of the three-dimensional structures in these designs is to resuspend particles into the mainstream flow by inducing turbulence with a high shear rate. Vortical cross-step filtration differs from these industrial crossflow designs by using specific structures that organize the separated shear layer and affect multiple features of sustained vortices [8], including the location, size, duration, rotational speed, tangential speed, and direction and speed of travel along the vortex axis. In turn, the vortices generated during cross-step filtration can suspend, concentrate, and transport particles along the filter [8].

Varying the angle of the backward-facing steps that formed *d*-type ribs in the 3D models (55°, 90°, or 110° with respect to the model midline) had significant effects on all four of the vortex parameters that were quantified (diameter, tangential speed, rotational speed, and speed of travel along the vortex axis within the slot, Table 1). Sanderson et al. [8] reported that the mass of brine shrimp cysts retained by models with these three rib angles did not differ significantly. These results indicate that, despite significant variation in vortex parameters and different patterns of particle deposition (Fig 5), models with these three rib angles presented approximately the same resistance to flow and filtered approximately the same volume of fluid per unit time. Given the extensive morphological diversity in the branchial arches and gill rakers of suspension-feeding fishes [1], the generation of vortices that differ in flow parameters and particle deposition patterns, as a consequence of evolutionary diversity in the branchial arch angles, deserves further study. The diameter, rotational speed, and tangential speed of the vortex determine the centripetal acceleration. Changes in rib angle that alter these vortex parameters could affect the movement of particles due to centripetal acceleration. Similarly, structural features of the *d*-type ribs other than rib angle (e.g., groove aspect ratio of fish branchial arches and gill rakers [8]) will have hydrodynamic effects with potential ecological and evolutionary impacts.

The vortex parameters that we quantified in the 3D physical models are very similar to those quantified here in the preserved paddlefish, indicating that the 3D models simulate structural features that are important for vortex generation in the preserved paddlefish. Our research has focused on the second gill slot of the 3D models and preserved paddlefish. While the trends reported here for vortex diameter and rotational speed can be generalized qualitatively to other slots in the 3D models, vortex parameters cannot be quantified as accurately in the first gill slot due to the greater turbulence of the separated shear layer over the “lip” of the 3D model and the dentary of the preserved paddlefish. In addition, slots 3–5 of the preserved paddlefish cannot be imaged completely through the open gape or reached with a thermistor flow probe (Fig 6). To quantitatively assess vortex parameters in slots 3–5, new techniques will have to be developed to allow thermistor flow probe measurements and flow visualization in the posterior oral cavity.

### Forced vortices

Our data on vortex parameters indicate that the vortices generated continuously downstream from backward-facing steps in the preserved paddlefish and the 3D models have the characteristics of forced (rotational) vortices rather than free (irrotational, potential) vortices [49]. Forced vortices are common at low to moderate Re (e.g., cavity flow and Moffatt eddies [50–52]; stable attached eddies generated by bluff bodies [53]). In a forced vortex, a continuous external source of kinetic energy must maintain the speed of the fluid throughout the vortex [54]. Fueled constantly by this external torque, a forced vortex rotates essentially as a solid (rigid) body with the outermost fluid traveling at a higher speed than the fluid that is closer to the vortex axis. The fluid circulates as a unit around the forced vortex axis, with the inner and outer streamlines completing the same number of revolutions in a given time. In a forced vortex, the speed of the fluid (tangential speed) is directly proportional to the distance from the axis of rotation of the vortex. Therefore, the angular velocity (Ω, s^-1^) is constant across the diameter of a forced vortex, and there is no shear within a forced vortex [27, 49]. Just as a turntable is a rotating solid body, the water in a beaker that rotates on a turntable will achieve solid-body rotation in the form of a forced vortex extending from the axis of the beaker to the outer walls. This forced vortex will then decay gradually when the turntable rotation ceases.

In a ram suspension-feeding fish, the kinetic energy of swimming is responsible for the flow of water into the oral cavity. In our flow tank, this kinetic energy was supplied by the motor and impeller, which maintained the speed of the water flowing past the stationary model in the tank. Water entered the gape of the preserved paddlefish and the 3D models, passed continuously over the medial faces of the backward-facing steps, and exited through the slots between the backward-facing steps. The speed of the water traveling directly above each backward-facing step determined the maximum flow speed that was possible in the forced vortex downstream from that rib. The boundary layer immediately above the medial face of each rib separated from the downstream edge of the rib and entered the slot downstream from the rib (Fig 1D). This separated shear layer, and any portion of the mainstream flow medial to the shear layer that also entered the slot [8], provided the energy for rotation of the forced vortex within the slot. In pump suspension-feeding species, the flow into the oral cavity caused by suction could provide the external torque for the rotation of forced vortices downstream from branchial arches, gill rakers, and/or denticles or branchiospinules during cross-step filtration.

**Fig 8:**
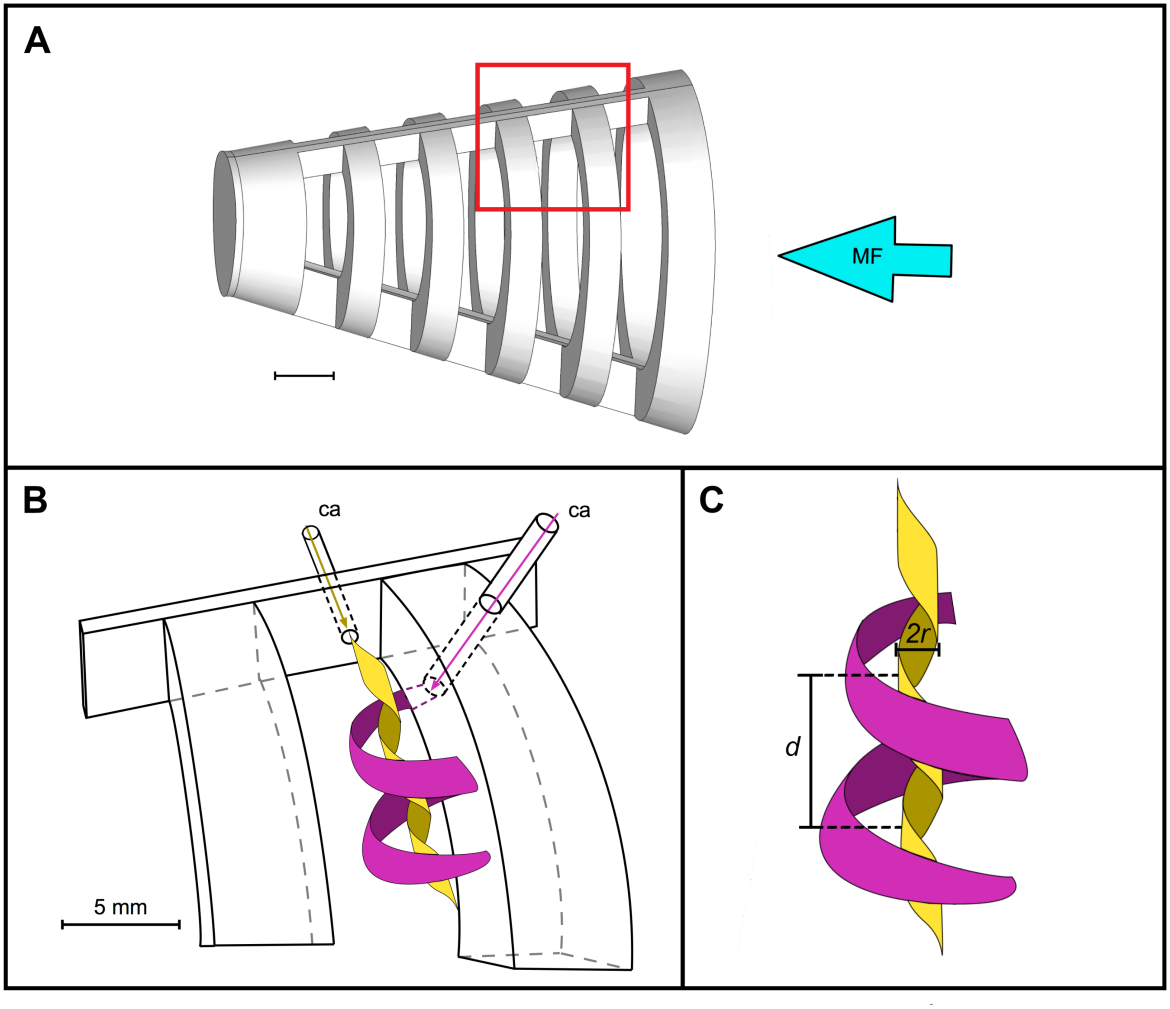
Inner and outer streamlines of the forced vortex visualized using water-tracing dye in the physical model. Red rectangle on the CAD image of the 90° model (A) denotes location of the enlargement (B) illustrating water-tracing dye released through cannulae (ca), with mainstream flow (MF) entering the open gape at the right of each image. Vortices are viewed from the exterior of the model, through the mesh, as in Fig 4. For clarity, the outer streamline of the forced vortex is shown in magenta and an inner streamline of the vortex is shown in yellow, with (C) illustrating the radius (*r*) and the distance (*d*) traveled by the yellow streamline along the slot during one complete revolution of the vortex. Scale bars 0.5 cm. Illustration by Eric Carstens; revised by M. Carly Lin.

Under specific circumstances, a vortex can be trapped and remain stable behind a backward-facing step [55–58]. A vortex that is not trapped can burst, causing substantial turbulence downstream [58, 59]. The scaling of the dorsal and ventral halves in our 3D models, as well as the addition of the simulated operculum, prevented the bursting of vortices through the mesh by trapping the vortices within the slot downstream from each rib. In the preserved paddlefish as well as in our 3D models to which a simulated operculum had been attached, we recorded a directed movement of each forced vortex along the vortex axis within each slot. The asymmetry of the paddlefish oral cavity and the 3D model design allowed more water to exit from the region of the ceratobranchial-epibranchial joints in the paddlefish and from the ventral region of the 3D models. Thus, by directing water to exit from specific regions, cross-step filtration can lead vortices to travel along their axes [8]. Under such circumstances, a forced vortex can travel along the vortex axis, creating helical streamlines [60]. In our study, the dye that tracked the helical streamlines of the vortices in the 3D models was released parallel to the vortex axis, near the vortex center, from a cannula that was flush with the slot wall along the dorsal midline of each model (Fig 4, yellow streamline in Fig 8). Thus, Table 1 presents data on inner streamlines of the vortices. In contrast, by releasing dye perpendicular to the vortex axis from a cannula that was flush with the medial face of the rib, Sanderson et al. [8] visualized the separated shear layer that entered the slot and formed the outer streamlines of the forced vortex (magenta streamline in Fig 8), farthest from the vortex center.

The inner helical streamlines of the vortices that we quantified in the 90° model had a mean diameter of 0.09 ± 0.02 cm and a mean tangential speed of 3.0 ± 0.8 cm s^-1^ (Table 1), whereas the outer helical streamlines of the vortices in this 90° model (reported in [8]) at the same flow tank speed had a mean diameter of 0.30 ± 0.02 cm and a mean tangential speed of 9.7 ± 0.6 cm s^-1^. Despite these differences in diameter, the mean rotational speeds of the streamlines closest to the vortex center *vs*. farthest from the vortex center were very similar (608 ± 45 rotations min^-1^ [Table 1] and 626 ± 68 rotations min^-1^ [8], respectively, n = 5 vortices). As discussed above, this constancy of rotational speed for the inner and outer streamlines is inherent in the solid-body rotation of a forced vortex (Fig 8). By definition, a forced vortex has a constant rotational speed throughout the vortex. Therefore, the speed of the fluid along any given streamline (tangential speed) must be directly proportional to the distance from the axis of rotation (i.e., directly proportional to the radius of the streamline). Thus, the solid-body rotation of the forced vortex explains the difference between the tangential speed of the inner *vs*. outer streamlines recorded in the 90° model. Similarly, solid-body rotation explains the comparable speed of travel along the vortex axis within the slot for the inner helical streamlines *vs*. the outer helical streamline in the 90° model (3.0 ± 0.4 cm s^-1^ [Table 1] and 3.0 ± 0.3 cm s^-1^ [8], respectively; n = 5 vortices).

The complexities of particle concentration and transport by vortices are dependent on the characteristics of both the vortex and the particle. The interactions between vortical flow characteristics, particle morphology, and particle motility have just begun to be explored [26–28, 61]. Theoretical and experimental models have been developed recently to quantify the non-intuitive interactions between small-scale turbulence and plankton. These studies have focused on the effects of vortex parameters on the movements and concentration of phytoplankton and zooplankton in coastal or near-surface zones [27–29]. Based on these studies, the forced vortices and separated shear layers in our 3D models and preserved paddlefish could have effects on the escape responses and concentration of motile phytoplankton and copepods. In addition, although the properties of the particles and the vortex determine whether inertial particles will be entrained or ejected by the vortex, these processes are not well understood [62]. Further research is needed on the highly complex three-dimensional movements of the forced vortices in the 3D models and the preserved paddlefish, as these vortices and the associated separated shear layers may be manipulated to suspend, concentrate, and transport particles [8].

### Fluid exit ratio for ram suspension-feeding fish

The ratio of the area for water to exit from the oral cavity divided by the area for water to enter through the gape of ram suspension-feeding fish, which we term the “fluid exit ratio”, has not been calculated previously for ram suspension-feeding fish or models [1, 39, 44]. The design of computational models and 3D physical models for the oral cavity of ram suspension-feeding fish requires decisions about oral gape area, gill slot area, and percent open pore area of the filtration surface. In the absence of a method to consider the potential combined effects of these morphological parameters on fluid flow through the oral cavity, previous models of ram suspension-feeding fish [39, 44] have been constructed by selecting values for each of these morphological parameters in isolation. Here, we have identified fluid exit ratio as a composite variable that can be used to inform the design of models for ram suspension-feeding fish and can be applied in future studies to assess how variation in oral gape area, gill slot area, and/or percent open pore area of the filtration surface affects filtration performance. For example, when the fluid exit ratio for a ram suspension-feeding fish decreases as the value for exit area is reduced below the value for entrance area, water will be increasingly diverted around the exterior of the open gape instead of entering the gape. Therefore, at a fluid exit ratio that is << 1, the values for vortex parameters and filtration performance will approach zero. At the other extreme, as the exit area is maximized and the fluid exit ratio increases above one during vortical cross-step filtration in a ram suspension-feeding fish, the backward-facing steps may be separated by increasingly wide slots and may resemble *k*-type ribs rather than *d*-type ribs [8]. Therefore, at a fluid exit ratio >> 1, water entering the gape may exit primarily from zone 1 (Fig 1D) where particles will clog the filter, and any small vortices that form will have minimal impact on filtration [8]. Future studies on filtration mechanisms in ram suspension-feeding fish can manipulate fluid exit ratio to investigate the relationships between morphological parameters, fluid exit ratio, vortex parameters, and swimming speed.

To calculate the numerator for the exit ratio, two components of the area for water to exit from the oral cavity must be known: (1) the summed area of the slots between the medial margins of the branchial arches (Table 4), and (2) the percentage of the slot area that is not blocked by gill rakers (i.e., the percent open pore area). In paddlefish that were preserved in suspension-feeding position, the summed slot area between the branchial arches was greater than the oral gape area (Table 4). Using published data on gill raker width measured midway between the gill raker base and tip in dead paddlefish, and the gap distance between gill rakers measured near the raker bases [63], we calculated that paddlefish with a size comparable to those studied here have gill rakers with an open pore area percentage of approximately 35%. This means that the summed area of the inter-raker spaces in those paddlefish was calculated to be 35% of the total area between the branchial arches. However, paddlefish gill rakers taper towards the tip [63], and paddlefish can control the abduction and adduction of the gill rakers across the slots between the branchial arches during ram suspension feeding [36, 64]. Therefore, the effective open pore area percentage during paddlefish ram suspension feeding could be greater than 35%, and we estimate that the effective fluid exit ratios for the three preserved paddlefish while alive, taking into account the blockage that would result from gill raker abduction in a suspension-feeding paddlefish, would be greater than 0.50–0.65.

For our 3D models of ram suspension-feeding fish, we used the area measured in SketchUp between the medial margins of the ribs, multiplied by the percent open pore area of the mesh, to calculate the numerator for the exit ratio. The total open pore area for water to exit from the models, divided by the total area for water to enter the models, resulted in an exit ratio of 1.6. When the 90° model was placed in a recirculating flow tank at realistic flow speeds, cross-step filtration occurred at very small intraoral pressures of approximately 11.5 Pa [8]. This filtration system at a low pressure head is consistent with the small intraoral pressures of approximately 13 Pa recorded during ram ventilation in live paddlefish [65] as well as the pressure head of 113 Pa calculated across the filtering apparatus of whale sharks [17].

A corollary of the small intraoral pressures in models of ram suspension-feeding fish is that, if the area available for water to exit from the model is less than the area available for water to enter the model (i.e., fluid exit ratio < 1), more of the water approaching the model is expected to be diverted around the exterior of the model gape rather than enter the model. Thus, when the exit ratio is less than one, the volume flow rate through the model may not be strictly proportional to the model gape area due to the formation of a substantial bow wave. Potential disadvantages of a larger bow wave anterior to the gape include a reduction in the volume of water filtered per unit time, the energetic cost of overcoming drag generated by the bow wave during forward locomotion, and the escape of prey that sense this flow disruption or that are diverted away from the gape by the bow wave [66–68]. Further research is needed to (1) calculate the fluid exit ratio for ram suspension-feeding species, (2) quantify intraoral pressure during ram suspension feeding, and (3) determine the relationship between fluid exit ratio, intraoral flow speeds, intraoral pressure, swimming speed, and the extent of the bow wave generated anterior to the gape during ram suspension feeding.

### Paddlefish branchial arch angles and head yaw angle

Previous models of ram suspension-feeding fish have chosen branchial arch angles of 55° and 90° with respect to the midline of the oral cavity [39, 44], but empirical measurements of these angles have not been available for fish with abducted branchial arches. Our measurements for paddlefish preserved in ram suspension-feeding position ranged from 26–46° for the anterior margins of the first and second gill slots, to 15–35° for branchial arches II-IV (Table 5). These measurements were used recently in a refined 3D-printed physical model of the juvenile paddlefish oral cavity during ram suspension feeding [33].

Head yaw [69] has been included in only one previous physical or computational model of ram suspension feeding [33]. We conducted flow tank experiments using preserved paddlefish positioned near the maximum yaw angle quantified from live paddlefish during ram suspension feeding [33]. Because head yaw changes the angle at which flow entering the oral cavity intersects all intraoral structures, head yaw also changes the angle at which the shear layer separates from the medial downstream edge of the branchial arch and enters the gill slot. Since the angle of the *d*-type ribs with respect to the midline of the roof in our 3D models had significant effects on all four of the vortex parameters that were quantified in our study (diameter, rotational speed, tangential speed, and speed of travel along the vortex axis within the slot), head yaw is also expected to affect vortex parameters.

## Conclusions

Vortices that are generated posterior of the branchial arches in 3D physical models and preserved paddlefish provide a novel mechanism for the suspension, concentration, and transport of particles in suspension-feeding fish. Our data on vortex parameters indicate that these vortices have the characteristics of forced vortices, with the flow of water inside the oral cavity providing the external torque required to sustain the vortex. We quantified the angles at which the backward-facing steps formed by branchial arches I–IV were arranged along the midline of the oral cavity roof and floor in the paddlefish that were preserved in ram suspension-feeding position. In our 3D physical models, the angle of the backward-facing step with respect to the model’s dorsal midline affected vortex parameters significantly, including diameter and rotational, tangential, and axial speed. We also identified a new variable for ram suspension feeding termed the fluid exit ratio (the ratio of the total open pore area for water leaving the oral cavity via spaces between branchial arches that are not blocked by gill rakers, divided by the total area for water entering through the gape during ram suspension feeding). The fluid exit ratio in preserved paddlefish was a significant predictor of the flow speeds that were quantified anterior of the rostrum, at the gape, directly dorsal of the first ceratobranchial, and in the forced vortex generated by the first ceratobranchial. Our data indicate that quantification of the fluid exit ratio should guide the design of future computational and physical models for ram suspension-feeding fish and has the potential to provide insight on vortical cross-step filtration. Understanding the factors that affect the operation of vortical cross-step filtration in ram suspension-feeding fish will require the development of new approaches and the quantification of previously unreported variables, including the fluid exit ratio and branchial arch angles during suspension feeding.
